# *Sphingomonas paucimobilis* - a rare cause of splenic abscesses: A case report

**DOI:** 10.1097/MD.0000000000028522

**Published:** 2022-01-07

**Authors:** Victoria Birlutiu, Simona Elena Dobritoiu, Andreea Magdalena Ghibu, Rares Mircea Birlutiu, Loredana Camelia Boicean

**Affiliations:** a Lucian Blaga University of Sibiu, Faculty of Medicine, Sibiu, Romania; b Academic Emergency Hospital Sibiu, Infectious Diseases Clinic, Sibiu, Romania; c Lucian Blaga University of Sibiu, Romania; d FOISOR Clinical Hospital of Orthopedics, Traumatology, and Osteoarticular TB, Bucharest, Romania; e Academic Emergency Hospital Sibiu, Sibiu, Romania.

**Keywords:** case report, *Sphingomonas paucimobilis*, splenic abscess

## Abstract

**Rationale:**

Infections with *Sphingomonas paucimobilis* are rarely described in the literature and can be community-acquired or associated with healthcare, especially in patients with chronic conditions (e.g., diabetes mellitus), malignancies, or other causes of immunosuppression, except in people without comorbidities. We present the case of a patient with diabetes mellitus and hypertension diagnosed during a routine evaluation, with splenic abscess caused by *S paucimobilis*. Our literature search revealed no other case report of splenic abscess caused only by *S paucimobilis*.

**Patient concerns:**

We present the case of a 55-year-old Caucasian man with type 2 diabetes mellitus and hypertension.

**Diagnosis:**

Thoraco-abdominal computed tomography revealed splenomegaly of 20X16X18 cm, with a homogeneous subcapsular hypodense collection, with a mass effect on the left hemidiaphragm.

**Interventions:**

The patient underwent surgical intervention and *S paucimobilis* was isolated on blood agar.

**Outcome:**

The patient received treatment with ciprofloxacin (500 mg twice daily) for 14 days, with favorable outcomes.

**Lessons:**

*S paucimobilis*, a low-virulence bacterium, can cause community-acquired or nosocomial infections. Visceral localizations, usually symptomatic, can evolve rapidly, and the diagnosis is associated with complications or, as in our case, with careful investigation of some changes in laboratory investigations.

## 1 Introduction

Infections with *Sphingomonas paucimobilis* are rarely described in the literature, with various localizations and varying degrees of severity, including sepsis,^[1]^ meningitis,^[2,3]^ endocarditis, visceral abscesses, enteritis, osteoarticular,^[4–6]^ urinary, skin, or soft tissue infections. Due to the reduced virulence of the bacteria, the infection is associated with either a patient with comorbidities, such as diabetes mellitus, chronic kidney disease, chronic respiratory diseases, liver cirrhosis, ethilism, or severe immunosuppression,^[1]^ exceptionally being associated with an immunocompetent host.

We present the case of a patient with diabetes mellitus and hypertension diagnosed during a routine evaluation, with splenic abscess caused by *S paucimobilis*.

## 2 Case report

We present the case of a 55-year-old Caucasian man, known to have type 2 diabetes and hypertension, with a stroke 5 years ago, without motor sequelae, former smoker, and alcohol user (up to 2 years ago), who was evaluated by a diabetologist who noticed an anemic syndrome and decided to conduct further laboratory and imaging investigations. The most important laboratory results are listed in Table 1.

**Table 1:** Dynamics of laboratory examinations.

Parameters	Day 1	Day 2	Day 4 (postoperatively)	Reference range
Leukocytes	16.9 × 10^3^		16.5 × 10^3^	3.60–10.5 × 10^3^/mm^3^
Hemoglobin	10.7		8.8	13–17 g/dL
Hematocrit	33		26.4	39%–50%
Medium cell volume	85.9		85.1	81–100 fL
Platelets	785		742	15–400 × 10^3^/mm^3^
Neutrophils	10.7 × 10^3^		12.4 × 10^3^	1.90–7.6 × 10^3^/mm^3^
Erythrocyte sedimentation rate	>100			0–15 mm/h
Fibrinogen		779.674		220–496 mg/dL
Serum iron	33			60–180 μg/dL
C-reactive protein		96.09		0–5 mg/L
Glycosylated hemoglobin	19.95			4%–6%
Glycaemia	214		218	70–115 mg/dL
Urea	64		39	15–45 mg/dL
Creatinine	0.96		0.66	0.6–1.2 mg/dL
Uric acid	8.27			3.50–7.20 mg/dL

**Figure 1:**
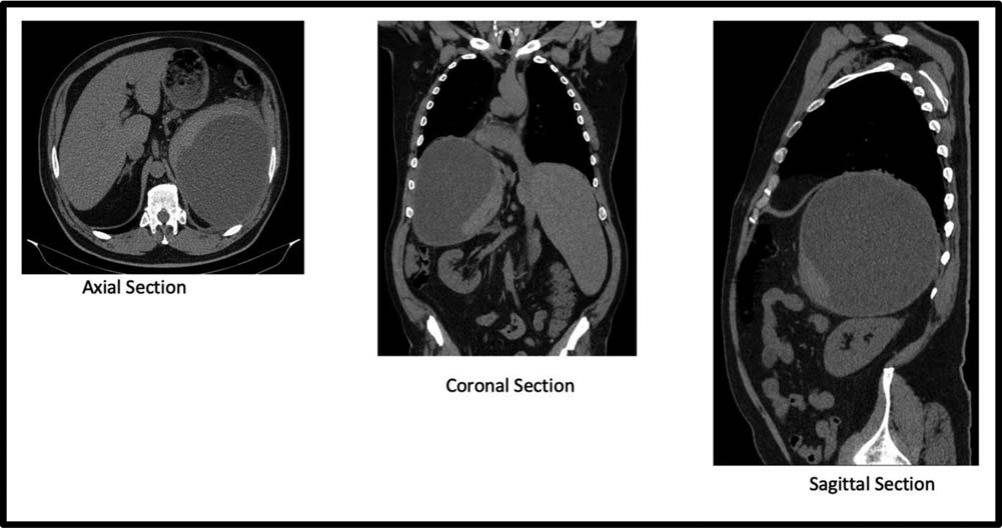
Thoracoabdominal computed tomography sections showing splenomegaly of 20 X16X18 cm, with a subcapsular hypodense collection, homogeneous fluid densities, mass effect on the left hemidiaphragm, compressive atelectasis, and focal pulmonary consolidation.

Abdominal ultrasound was performed, which suggested a possible splenic hematoma, and subsequently, computed tomography was recommended. Thoracoabdominal computed tomography revealed the following pathological changes: splenomegaly of 20X16X18 cm, with a subcapsular hypodense collection, homogeneous fluid densities (5–14 Hounsfield units), mass effect on the left hemidiaphragm, compressive atelectasis, and focal pulmonary consolidation (Fig. 1).

The patient worked in a warehouse with building materials, had no previous contact with medical services, and did not describe any recent change in health.

Surgery was performed and confirmed intra-operatively with a splenic abscess; splenectomy and bacteriological examination of the pus were performed. All the biological samples were inoculated and incubated aerobically and aerobically at 37°C. The isolated bacteria were identified using a VITEK 2 Compact analyzer (bioMérieux, Marcy-l’Étoile, France). Minimum inhibitory concentrations were assessed according to the European Committee on Antimicrobial Susceptibility Testing breakpoints. *S paucimobilis* was isolated from blood agar. The identified strain was sensitive to cefoxitin, ceftazidime, ciprofloxacin, gentamicin, meropenem, nitrofurantoin, and norfloxacin, and resistant to cotrimoxazole. The patient received treatment with ciprofloxacin (500 mg twice daily) for 14 days, with favorable outcomes.

## 3 Discussions

*S paucimobilis* (also called *Pseudomonas paucimobilis*) is a yellow-pigmented gram-negative bacillus that is aerobic, catalase-positive, oxidase-positive, flagellate, and unsporulated. It has low virulence, motivated by the lack of lipopolysaccharide A,^[7,8]^ replaced by glycosphingolipid, which triggers the release of proinflammatory cytokines in the body, which is 10 times lower than that induced by lipopolysaccharide A.^[1]^*S paucimobilis* is ubiquitous, present on the ground, in water, and also in a hospital environment, and can contaminate distilled water or other sterile solutions, respiratory devices, or those used in hemodialysis services. Infections with *S paucimobilis* can be community-acquired or associated with healthcare, especially in patients with chronic conditions (e.g., diabetes mellitus), malignancies, or other causes of immunosuppression,^[9]^ except in people without comorbidities.

Cases of meningitis with *S paucimobilis* are cited in the literature,^[2,3]^ as well as a case associated with external ventricular drainage.^[10]^*S paucimobilis* has been identified in bacteremia, sepsis, and septic shock.^[1]^ Cases of septic arthritis, osteomyelitis,^[4–6]^ septic pulmonary embolism,^[5]^ peritonitis associated with peritoneal catheterization, postoperative endophthalmitis,^[11]^ brain abscesses, and skin and soft tissue infections,^[12,13]^ are described. Ventilation pneumonia,^[14]^ adenitis, myositis,^[15]^ urinary tract infections, acute enteritis,^[16]^ and abdominal abscesses associated with peritonitis have also been reported.^[17]^

In only 1 case published in 1987, *S paucimobilis* was identified in a splenic abscess associated with sepsis caused by *Clostridioides difficile*.^[18]^ Our literature research reported no other case report of splenic abscess caused by *S paucimobilis*.

Lin et al^[1]^ identified 16 cases of bacteremia caused by *S paucimobilis* in Taiwan between 2004 and 2008. Taking into consideration the data from the literature, the authors described the predominance of cases in men (57.1%), with a median age of 48.5 years. Most cases were identified in patients with malignancies (57.1%), under immunosuppressive therapy (40.5%) and patients with diabetes in 11.9% of cases; ethilism and liver cirrhosis were associated with bacteremia with *S paucimobilis* in 9.5% of cases; 7.1% were patients with end-stage renal disease and chronic respiratory disease was identified in 4.8% of patients. 69% of the cases were healthcare-associated infections, particularly those associated with central venous catheterization (33.3%). The evolution toward healing in all the cases reported by Lin (42, including 3 cases of infectious shock) once again supports the low virulence of *S paucimobilis*.

The case presented by us occurred in a patient without contact with medical services, without parenteral therapies or invasive examinations, but who had diabetes, hypertension, and a history of stroke.

Perola et al^[19]^ describe the presence of cases associated with water contamination in a hematological unit, with recurrent infection in a patient with leukemia, and Mayberry et al^[20]^ identified the transmission of *S paucimobilis* in an oncology service through improper handling and improper dilution, with non-sterile water, of analgesics (syringes with hydromorphone).

Antibiotic susceptibility is generally preserved for third-generation cephalosporins, beta-lactamine-beta-lactam combinations, carbapenems, aminoglycosides, and fluoroquinolones, although cases of local resistance are possible. In a series of 16 patients with bacteremia presented by Lin, over 80% of the strains retained their susceptibility to fluoroquinolones, levofloxacin, ciprofloxacin, betalactamines-betalactamase inhibitors, ampicillin/sulbactam, piperacillin/tazobactam, and imipenem.

Peel et al^[13]^ isolated from skin ulcer a strain sensitive to most aminoglycosides (except streptomycin), tetracycline, cotrimoxazole, and chloramphenicol but resistant to polymyxin, cephalothin, and nalidixic acid.

The strain isolated from the splenic abscess in the present case was sensitive to cefoxitin, ceftazidime, ciprofloxacin, gentamicin, meropenem, nitrofurantoin, and norfloxacin, but resistant to cotrimoxazole. Treatment with ciprofloxacin (1 g/day) for 14 days was indicated according to the susceptibility tests, with good tolerance and without side effects. Prophylaxis through vaccination for pneumococcal, meningococcal, and *Haemophilus influenzae* type b infections was recommended to the patient.

## 4 Conclusions

*S paucimobilis*, a low-virulence germ, can cause community-acquired or nosocomial infections, especially in patients with chronic conditions or immunocompromised patients.

Visceral localizations, usually symptomatic, can evolve rapidly, and the diagnosis is associated with complications or, as in our case, with careful investigation of some changes in laboratory investigations.

## Author contributions

VB, RMB, and LCB contributed equally to this manuscript in terms of the acquisition, analysis, and interpretation of data, conception and design, and drafting of the manuscript. VB, SD, and AG were involved in providing treatment for the patient and collecting the data. VB, LCB, and RMB drafted the manuscript. All the authors have been involved in revising the manuscript. All authors have read and approved the final version of the manuscript.

**Conceptualization:** Victoria Birlutiu, Rares Mircea Birlutiu, Loredana Camelia Boicean.

**Data curation:** Victoria Birlutiu, Simona Elena Dobritoiu, Andreea Magdalena Ghibu, Rares Mircea Birlutiu, Loredana Camelia Boicean.

**Formal analysis:** Victoria Birlutiu, Andreea Magdalena Ghibu, Rares Mircea Birlutiu, Loredana Camelia Boicean.

**Investigation:** Victoria Birlutiu, Simona Elena Dobritoiu, Andreea Magdalena Ghibu, Rares Mircea Birlutiu, Loredana Camelia Boicean.

**Methodology:** Victoria Birlutiu, Rares Mircea Birlutiu, Loredana Camelia Boicean.

**Supervision:** Victoria Birlutiu, Rares Mircea Birlutiu, Loredana Camelia Boicean.

**Validation:** Victoria Birlutiu, Rares Mircea Birlutiu, Loredana Camelia Boicean.

**Visualization:** Victoria Birlutiu, Rares Mircea Birlutiu, Loredana Camelia Boicean.

**Writing – original draft:** Victoria Birlutiu, Rares Mircea Birlutiu, Loredana Camelia Boicean.

**Writing – review & editing:** Victoria Birlutiu, Rares Mircea Birlutiu, Loredana Camelia Boicean.
